# Evaluating Decision Support Tools for Precision Nitrogen Management on Creeping Bentgrass Putting Greens

**DOI:** 10.3389/fpls.2022.863211

**Published:** 2022-05-19

**Authors:** Qiyu Zhou, Douglas J. Soldat

**Affiliations:** Department of Soil Science, University of Wisconsin-Madison, Madison, WI, United States

**Keywords:** turfgrass, precision nitrogen management, decision support tool, nitrogen use efficiency, machine learning, random forest

## Abstract

Nitrogen (N) is the most limiting nutrient for turfgrass growth. Few tools or soil tests exist to help managers guide N fertilizer decisions. Turf growth prediction models have the potential to be useful, but the lone turfgrass growth prediction model only takes into account temperature, limiting its accuracy. This study investigated the ability of a machine learning (ML)-based turf growth model using the random forest (RF) algorithm (ML-RF model) to improve creeping bentgrass (*Agrostis stolonifera*) putting green management by estimating short-term clipping yield. This method was compared against three alternative N application strategies including (1) PACE Turf growth potential (GP) model, (2) an experience-based method for applying N fertilizer (experience-based method), and (3) the experience-based method guided by a vegetative index, normalized difference red edge (NDRE)-based method. The ML-RF model was built based on a set of variables including 7-day weather, evapotranspiration (ET), traffic intensity, soil moisture content, N fertilization rate, NDRE, and root zone type. The field experiment was conducted on two sand-based research greens in 2020 and 2021. The cumulative applied N fertilizer was 281 kg ha^−1^ for the PACE Turf GP model, 190 kg ha^−1^ for the experience-based method, 140 kg ha^−1^ for the ML-RF model, and around 75 kg ha^−1^ NDRE-based method. ML-RF model and NDRE-based method were able to provide customized N fertilization recommendations on different root zones. The methods resulted in different mean turfgrass qualities and NDRE. From highest to lowest, they were PACE Turf GP model, experience-based, ML-RF model, and NDRE-based method, and the first three methods produced turfgrass quality over 7 (on a scale from 1 to 9) and NDRE value over 0.30. N fertilization guided by the ML-RF model resulted in a moderate amount of fertilizer applied and acceptable turfgrass performance characteristics. This application strategy is based on the N cycle and has the potential to assist turfgrass managers in making N fertilization decisions for creeping bentgrass putting greens.

## Introduction

Well-managed turfgrass systems that include golf courses can provide many beneficial environmental services to our society (Lonsdorf et al., 2021). However, natural resources, which include soil, water, and air, are negatively affected by the intensive use and production of agrochemicals. In turfgrass systems, especially on the highly maintained golf courses and athletic fields, agrochemicals are used to achieve desired aesthetics and functions. Nitrogen (N) fertilizer is applied in amounts greater than all other nutrients. US golf courses used 55,333 Mg N fertilizer annually (Gelernter et al., 2016). These N inputs pose potentially significant nonpoint source pollution risks (Bock and Easton, 2020). Optimizing the N application rate is one of the most effective ways to improve turfgrass management and reduce its potentially negative environmental impacts.

Maintenance of a high-quality playing surface of a golf course takes priority over maximizing turfgrass yield as is common for agricultural crops. Specifically, golf course putting greens are the main focus for most golf course managers (Hammond and Hudson, 2007); therefore, putting greens usually receive the most resource inputs and energy use per unit area (Gelernter et al., 2016). N is the most limiting nutrient for turfgrass, is an important driver of plant growth, and plays an important role in the visual quality of the surface. Relatively high N fertilization rates result in verdant and aesthetically pleasing playing surfaces. However, the rapid growth induced by relatively high N fertilization increases thatch and soil organic matter which reduces the function (e.g., ball roll speed) and aesthetics of putting greens (Meinhold et al., 1973; Murray and Juska, 1977; Throssell, 1981; Gaussoin et al., 2013). On the other hand, putting greens receiving relatively low rates of N fertilization can be slow to recover from ball marks and wear damage from foot traffic which encourages weed invasion (Beard, 1972).

Other nutrient application decisions, such as potassium, phosphate, calcium, magnesium, and so on., can be guided by soil testing (Murphy and Murphy, 2010; Landschoot, 2017). However, most commercial soil testing laboratories do not offer tests for estimating available N in soil. Tests for N exist, but they are often not quick nor cost-effective. Furthermore, plant-available N in the soil is affected by weather, so the N release pattern varies during the growing season. Lacking a tool or test, N application recommendations for golf courses putting greens are often based solely on turfgrass managers' experience and observations of turf quality. Annual recommended N fertilization amounts for golf course putting greens generally range from 49 to 195 kg ha^−1^ y^−1^ (Murphy and Murphy, 2010; Landschoot, 2017). Applying N fertilizer based on turfgrass visual performance might be warranted for turfgrass showing signs of inadequate N such as chlorosis, decreased density and growth, and slower recovery from abiotic and biotic stresses. However, golf turf managers prefer to avoid these negative responses and therefore regularly make fertilization applications to turfgrass that is performing optimally. This could result in overapplication because optimally performing turfgrass may perform well with optimum and above-optimum N. It is clear that a more objective N application strategy is needed to maximize N fertilizer efficiency.

Turfgrass visual quality assessment has been widely used as a standard to evaluate turfgrass response to various management practices. It involves a subjective visual evaluation of a turfgrass stand on a scale of 1 to 9 (where 1 represents completely dead turf, 6 represents the minimally acceptable quality, and 9 represents ideal turfgrass quality) based on the evaluator's mental integration of turfgrass color, uniformity, and shoot density (Beard, 1972). With the recent development and increasing availability of sensor technology, turfgrass professionals are able to utilize spectral reflectance data obtained from proximal and remote sensors to subjectively quantify turfgrass response to various practices, including N fertilization. Spectral reflectance is measured with given wavelengths of light, and studies (Trenholm et al., 1999; Bell et al., 2002; Fitz–Rodríguez and Choi, 2002; Keskin et al., 2008) have shown that spectral reflectance could be well correlated with visual quality for turfgrass species maintained under different management practices. Spectral reflectance is sensitive to N fertilization of turfgrasses (Caturegli et al., 2016; Guillard et al., 2016) and therefore has the potential to serve as an objective measurement of turfgrass performance. For example, spectral reflectance has been used to detect chlorophyll concentration and has also been shown to have a good correlation with plant N status (Horler et al., 1983; Steven and Clark, 2013). However, few studies have evaluated the feasibility of making N application decisions solely based on spectral reflectance measurement of turfgrass on golf course putting greens, and these measurements are likely to play a larger role in the precision management of turfgrass in the future.

Precision turfgrass management aims to provide optimal management of pests, fertilizer, salinity, cultivation, and irrigation (Stowell and Gelernter, 2006; Carrow et al., 2007; Bell and Xiong, 2008; Krum et al., 2010). Precision N management is a branch of precision turfgrass management that seeks to match N supply with turfgrass N demand spatially and temporally to maximize turfgrass function and minimize nutrient loss from the turf system. On sand-based putting green soils, the N cycle can be simplified using a few assumptions. Potential N loss by denitrification, volatilization, runoff, and leaching is expected to be negligible or quite low when best management practices are used (Snyder et al., 1984; Morton et al., 1988; Gross et al., 1990; Miltner et al., 1996; Erickson et al., 2001, 2008). This leaves clipping removal as the primary output of N, and fertilization as the primary input (assuming negligible input from irrigation water sources and atmospheric deposition). Moreover, Zhou and Soldat (2021a) concluded that tissue N of putting green creeping bentgrass spanned 2.5 and 5% during the growing season under typical conditions with an average of 3.9%. The optimal N fertilization rate (input) can be estimated by quantifying the N outputs (clipping mass x clipping tissue N concentration). This requires accurate estimates or measurements of turfgrass clipping yield, so that optimal N fertilizer inputs can be estimated.

The turfgrass growth potential (GP) model was developed by Gelernter and Stowell (2005) to aid decision-making related to fall overseeding on golf courses. Later, the turfgrass GP model was recognized as a tool for determining monthly or annual turfgrass N requirements based on the turfgrass growth potential (Woods, 2013), and this tool is used by turfgrass managers to guide N fertilization application decisions. The turfgrass GP model uses average air temperature to estimate turfgrass growth potential which spans 0 to 100%. For cool-season grasses, the model assumes that the optimal average air temperature for growth is 20°C (growth potential = 100%); as the temperature deviates from 20°C, the growth potential will decrease correspondingly until the growth potential reaches 0% at 0 and 40°C. The turfgrass GP model assumes that N should be applied to match the turfgrass' growth potential. An obvious pitfall with this method is that turfgrass growth is determined by complex physiological processes including genetic potential, environmental, and edaphic factors in addition to air temperature. For example, foot traffic stress, which is one of the most common stresses on golf course putting greens (Beard, 1972; Carrow and Martin Petrovic, 1992), can result in turf damage and reduced turf quality and clipping yield (Shearman et al., 1974; Shearman and Beard, 1975; Carrow and Martin Petrovic, 1992; Bilgili and Acikgoz, 2007). Water availability is another important factor of turfgrass growth. Limited access to water results in reduced root growth (Beard and Daniel, 1965), and excessive irrigation has been shown to also be detrimental to turfgrass growth and visual quality (Beard, 1972; DaCosta and Huang, 2006). Although the turfgrass GP model is useful for understanding how temperature may influence growth across regional or larger scales, at the local scale, a more detailed model could be useful for making more accurate predictions of turfgrass growth and corresponding N need.

In precision agriculture, crop growth models are widely used to guide N applications based on crop N demand to ensure increased N use efficiency. These models often estimate crop N needs by accounting for soil-plant processes, environmental conditions, and interactions with various management practices. A common approach for building a crop growth model is to use historical data to empirically predict future crop yield *via* machine learning (ML) (Jaikla et al., 2008; Brdar et al., 2011; Fukuda et al., 2013; Kuwata and Shibasaki, 2015; Everingham et al., 2016; Zhang et al., 2019; Van Klompenburg et al., 2020). Because of the differences in managing agriculture crops compared to turfgrass as well as the differing goals between agricultural production and turfgrass management, the utility of ML to aid turfgrass management decisions needs to be tested. Zhou and Soldat (2021b) developed turfgrass growth models with an ML approach and reported that the random forest (RF) algorithm was the best among those algorithms that tested for predicting creeping bentgrass clipping production on golf course putting green. The ML-RF turfgrass growth model inputs included daily weather, evapotranspiration (ET), soil moisture content, number of rounds of play, root zone type, N fertilization inputs, and NDRE. The aims of this study were to (1) evaluate the feasibility of the turfgrass ML-RF growth prediction model for improving N management and (2) compare how various N application strategies and decision support tools in terms of N applied and turfgrass performance characteristics. Specifically, the study evaluated two turfgrass growth models for guiding N fertilization: the PACE Turf growth potential model and an ML-RF growth prediction model against two more traditional approaches to N fertilizer management: the standard experience-based approach (which is the current standard for putting green fertilization) and an experience-based approach modified by reflectance measurements.

## Methods and Materials

### Study Sites

The study was conducted at the University of Wisconsin-Madison O.J. Noer Turfgrass Research and Education Facility located in Verona, WI, USA. Field experiments were conducted on two different sand-based putting green root zones in 2020 and 2021 using the same plots in each year. Both research greens were constructed according to USGA recommendations (U.S. Golf Association, 2004) in 2000. Root zone characteristics are reported in Table 1. Both greens were established in 2011 with “Focus” creeping bentgrass (*Agrostis stolonifera*), which is the most commonly planted cool-season grass species used on golf courses putting greens in this region. Research plots were irrigated daily to replace 70% of reference ET as estimated by an on-site weather station. The research greens were topdressed with 0.6 m^3^ ha^−1^ of sand approximately every 3 weeks during the growing seasons. Hollow tine cultivation was conducted once near the end of each growing season (September), and the cores were removed and holes filled with topdressing sand. Disease and other pests were monitored and controlled as needed. A total of four N treatments along with a non-fertilized control treatment were imposed on both root zones, and a detailed description of the four treatments can be found in Section Nitrogen Application Strategies. The experiment was set out as a completely randomized design with four replicates, and each plot measured 1.2 m by 2.4 m.

**Table 1:** Soil chemical properties of two putting green root zones.

**Root zones ID**	**Depth**	**SOM ^Θ^**	**P ^ϕ^**	**K**	**Ca**	**Mg**	**CEC ^ζ^**	**pH**
	**(cm)**	**(g kg^−1^)**	**(mg kg^−1^)**				**(cmol kg^−1^)**	
A	0–5	0.7	25.9	40.7	586.6	133.1	3.0	7.7
	5–10	0.5	24.1	17.2	429.9	101.6	3.0	7.5
B	0–5	1.2	64.2	91.6	1210.0	295.8	8.0	7.5
	5–10	0.6	17.0	25.5	578.5	143.6	4.0	7.3

θ *SOM, soil organic matter by loss on ignition (360°C for 2 h) (Davies, 1974).*

ϕ *Nutrients extracted via Mehlich-3 (Mehlich, 1984).*

ζ *CEC, cation exchange capacity via summation of extracted cations*.

### Turfgrass Data Collection

Clipping was collected from both research greens during 2020 and 2021 approximately every other day between 9:00 and 12:00 (weather permitting) by mowing a 1.9 m pass down the center of each plot using a 0.54-m-wide walking greens mower (Toro Co., Bloomington, Minnesota, USA). Before each clipping collection, 0.27-m wide alleys were mowed at the top and bottom of each plot perpendicular to the collection pass. This was done to reduce the variability associated with starting and stopping the mower. The effective clipping collection area for each plot was 1 m^2^. Turfgrass was maintained at 3.2 mm during the research period for both research greens. Clippings were brushed from the mower bucket into paper bags, which were then placed in an oven set to 50°C for at least 48 h. Sand and other debris were removed from the dried clippings using the water method described in Kreuser et al. (2011). NDRE of each plot was also recorded prior to each clipping collection event. In this study, to quantify the estimated N uptake of creeping bentgrass, we assumed that the tissue N concentration was 3.9% throughout the research.

### Nitrogen Application Strategies

#### Traditional N Fertilization Plan (Experience-Based)

Traditionally, N fertilization rates are based on manager experience, observations, and recommendations from local services or organizations. In this region, golf course putting greens typically receive between 100 and 250 kg N ha^−1^ y^−1^. At the O.J. Noer Turfgrass Research Facility, it would be typical for the station manager to apply 10 kg ha^−1^ every other week during the ~30-week growing season to putting green plots, for a total of ~150 kg ha^−1^ N per season. Therefore, for this study, the traditional N fertilization plan treatment utilized a 10 kg ha^−1^ application every other week during the growing season, which approximately spanned the period of May to October. Urea was used as the N source and was dissolved and sprayed as a liquid at a nozzle pressure of 40 psi. using a CO_2_ pressurized boom sprayer equipped with two XR Teejet 8004 VS nozzles.

#### Turfgrass Vegetative Index-Guided N Fertilization Method (NDRE-Based)

Another N treatment in the study used the normalized difference red edge (NDRE) obtained from the handheld proximal sensor (Rapid SCAN CS-45, Holland Scientific Inc., Lincoln, NE) to guide N application. NDRE is calculated using the combination of near-infrared red light (±800 nm) and red-edge band (±720 nm). NDRE is designed for crops with relatively high canopy density because the red edge band is able to penetrate deeper through the plant canopy. To calibrate the sensor and use spectral reflectance to guide N fertilization, the spectral reflectance readings from the turfgrass area are compared with the readings from reference strips, and then, fertilizer decisions are made according to the relationship between the two readings (Blackmer and Schepers, 1995; Raun et al., 2008; Samborski et al., 2009; Holland and Schepers, 2013; Guillard et al., 2021). One of the types of reference strips for spectral reflectance is called the virtual reference concept (Holland and Schepers, 2013). This method requires obtaining the spectral reflectance references from uniform research areas in the field where the turfgrass looks the greenest (well-fertilized) and the least green (under-fertilized) by visual observation. Based on the relationship between NDRE and N rate of well-fertilized and under-fertilized turf, a N fertilizer recommendation for turfgrass would be made based on the NDRE reading of an unknown area. In the study, we followed a similar approach but with some adjustments. Instead of finding the greenest and least green strip, we aimed to maintain the turfgrass at a minimally acceptable visual turfgrass quality. Therefore, the reference strips were the turfgrass research areas at a visual quality of ~6. The mean NDRE on turfgrass areas with a quality of 6 was 0.28. Therefore, if the mean NDRE of the treatment for the preceding 14 days was > 0.28, and then, no additional N fertilizer was added; otherwise, additional N fertilizer was applied at 10 kg ha^−1^. NDRE was collected by scanning the research plot approximately 1 m above the canopy and was measured three times each week during the growing season prior to each clipping collection.

#### Growth Potential (GP) Model (PACE Turf GP Model)

The GP model was presented as equation (1) (Woods, 2013). N fertilization for this treatment was applied every other week (consistent with all other treatments in this study), and the N application rate was determined by the GP (which is a percentage) multiplying by a maximum daily N use rate and multiplying that by the number of days since the last fertilization event. For this study, the maximum daily N use rate was determined to be 3.2 g m^−2^ d^−1^ based on growth and tissue N data collected on the same root zone during the 2019 growing season. This translates to a maximum N fertilization amount of 17.5 kg ha^−1^ every 2 weeks. The amount of N applied to each event was corrected by the GP of the previous 14 days.


(1) $$\begin{array}{l} GP=\frac{1}{e^{0.5{\left(\frac{T-T_{0}}{var}\right)}^{2}}} \end{array}$$

where

e: 2.718

T: local daily average temperature; °C

T_0_: optimal temperature for turfgrass growth; 20°C for cool-season grass

Var: adjust the change in GP as temperature moves away from T_0_; 5.5 for cool-season grass.

#### Machine Learning-Random Forest (ML-RF) Model

For the final N treatment, we used an RF algorithm for predicting turfgrass clipping yield using ML (Zhou and Soldat, 2021b). The model was constructed using clipping yield data collected in 2019 and 2020, where clipping data collected in 2019 were used to train the model to predict 2020's turfgrass clipping production, and clipping yield data collected in 2019 and 2020 were used to train the model to predict the 2021 turfgrass clipping production. Variable inputs when making predictions included (1) 3-day average soil moisture content (%) which was the average soil moisture content on the clipping collection event and the one before, soil moisture content was measured by time-domain reflectometry with 7.6-cm rods (FieldScout TDR 350, Spectrum Technologies, Aurora, Illinois, USA); (2) average weekly traffic intensities (round wk^−1^); (3) NDRE; (4) categorical value representing the root zone of the two putting greens; (5) days between mowing events; (6) daily weather variables obtained from the nearest weather station reported on Weather Underground (an open online real-time weather information website). These variables included daily maximum temperature (Tmax) (°C), minimum temperature (Tmin) (°C), average temperature (Tavg) (°C), precipitation (precip) (mm), maximum relative humidity (RHmax) (%), minimum relative humidity (RHmin) (%), average relative humidity (RHavg) (%), and average wind speed (Windavg) (km h^−1^); and (7) ET (mm) from a local weather station. A detailed description of the processes of choosing the input variables and detail about building and validating the model was presented by Zhou and Soldat (2021b).

**Figure 1:**
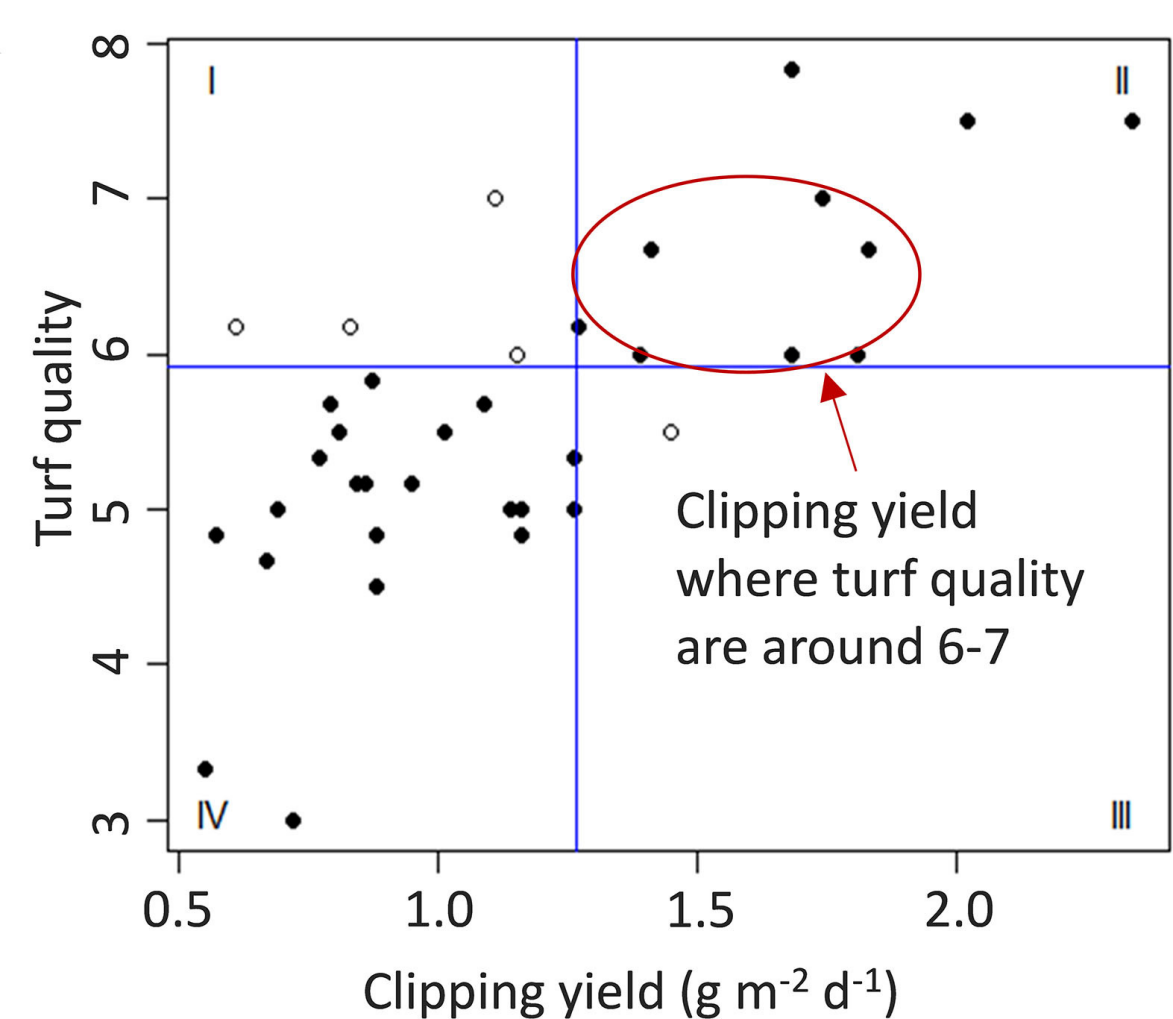
Relationship between creeping bentgrass dry clipping yield and turf quality where the clipping and turf quality were collected in 2018 at the University of Wisconsin–Madison turfgrass research facility, Verona WI, USA. Turf quality was evaluated on a scale from 1 to 9 where 1 represents completely dead turf, 6 represents the minimally acceptable quality, and 9 represents ideal turfgrass quality.

The goal of using ML-RF growth model to guide N application was to accurately predict turfgrass clipping production and then make N fertilization decisions to maintain the clipping production within a target range. As maintaining a good quality of turfgrass is still the ultimate goal of turfgrass management, target clipping production was determined by making observations about the relationship between clipping production and turf quality (Figure 1). We selected our target clipping production range to coincide with visual turfgrass quality between 6 and 7, which required the daily clipping production to be between 1.25 and 1.6 g m^−2^ d^−1^. The N fertilization decision used the following logic: (1) if the predicted 2-week cumulative clipping production was between 17.5 and 22.5 g m^−2^, the N fertilization rate would replace the N removed as estimated by the model predicted clipping yield multiplied by the estimated average tissue N concentration (3.9%); (2) if the predicted clipping yield was > 17.5 g m^−2^ 2 wks^−1^, then the N fertilization rate was determined using the median clipping yield (20 g m^−2^ 2 wks^−1^) of the ideal clipping removal range multiplied by the estimated average tissue N concentration (3.9%). If the model predicted clipping yield in excess of 22.5 g m^−2^ for a given 2-week period, then no N fertilizer would be added. However, this only occurred one time during the study period (July 2021), and the predicted clipping yield was in the range of 17.5 to 22.5 g m^−2^ 2 wks^−1^ for the majority of the study except at the beginning of each year's data collection, where the estimated clipping yield was lower than the target range.

### Statistical Analysis

Statistical analysis was conducted using ANOVA for clipping yield, NDRE, turfgrass quality, cumulative clipping yield overall 2 years and N use efficiency (NUE) using JMP software (version 15.0, SAS Institute Inc., USA). Data collected from 2020 to 2021 were pooled and analyzed collectively. Years were considered as a random effect in this study. Treatment means were separated using Fisher's protected least significant difference (LSD) using a *p*-value of 0.05. Box plots were made to represent the distribution of the ratio of predicted clipping and actual clippings. The box plots were set at maximum values, 75th (the upper quartile), median, 25th (the lower quartile), and minimum. Pearson correlation was conducted to quantify the correlation between clipping yield and NDRE.

## Results

**Figure 2:**
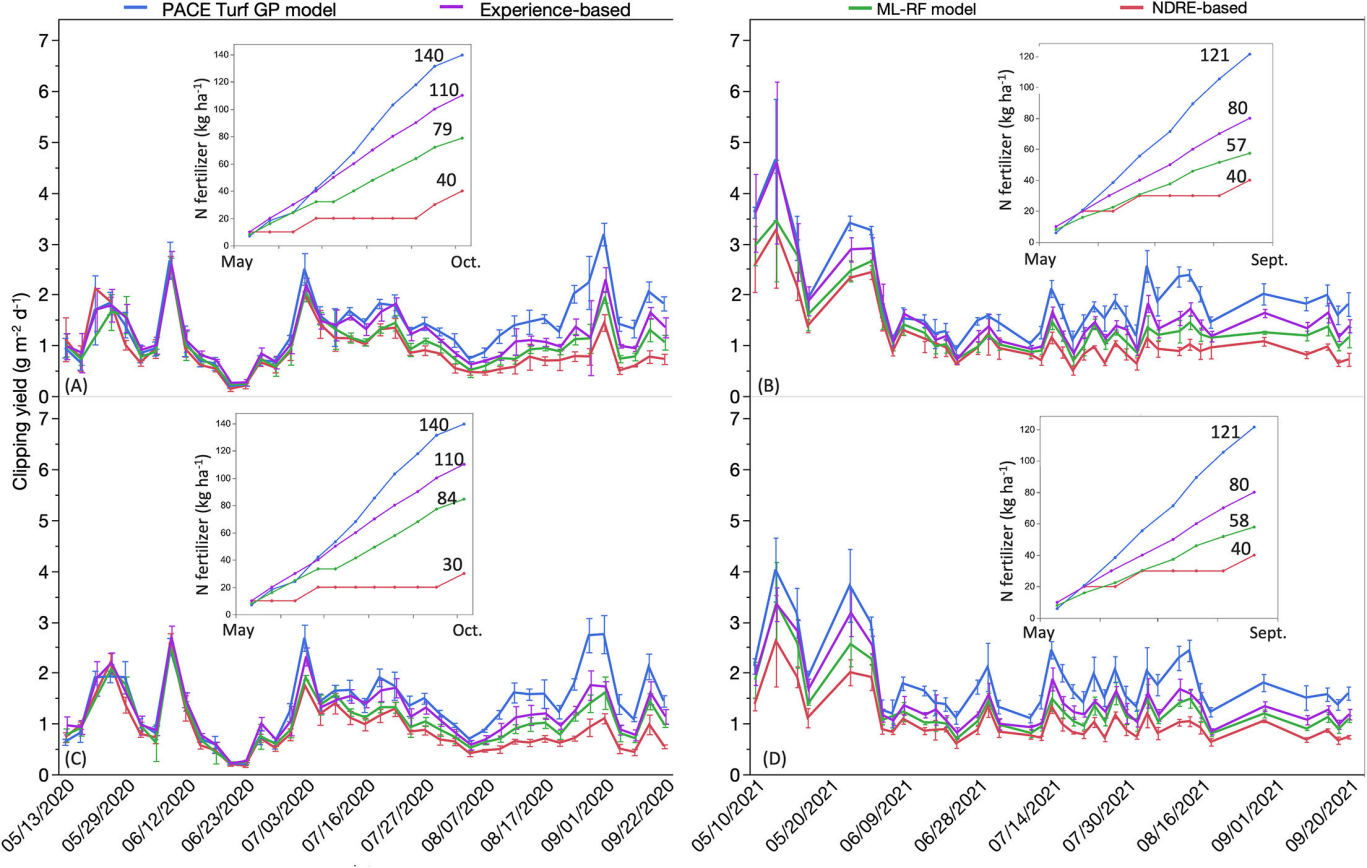
Creeping bentgrass clipping yield response to four nitrogen (N) application strategies, which include PACE Turf growth potential (GP) model, traditional N fertilization plan (experience-based), machine learning-random forest (ML-RF) method, and vegetative index-based strategy (NDRE-based). **(A)** creeping bentgrass growth response on root zone A, data collected in 2020. **(B)** bentgrass growth response on root zone A, data collected in 2021. **(C)** bentgrass growth response on root zone B, data collected in 2020. **(D)** bentgrass growth response on root zone B, data collected in 2021. Inserted figures in each panel represent the cumulative N fertilizer usage for each year on each root zone. Error bars indicate standard deviation.

**Figure 3:**
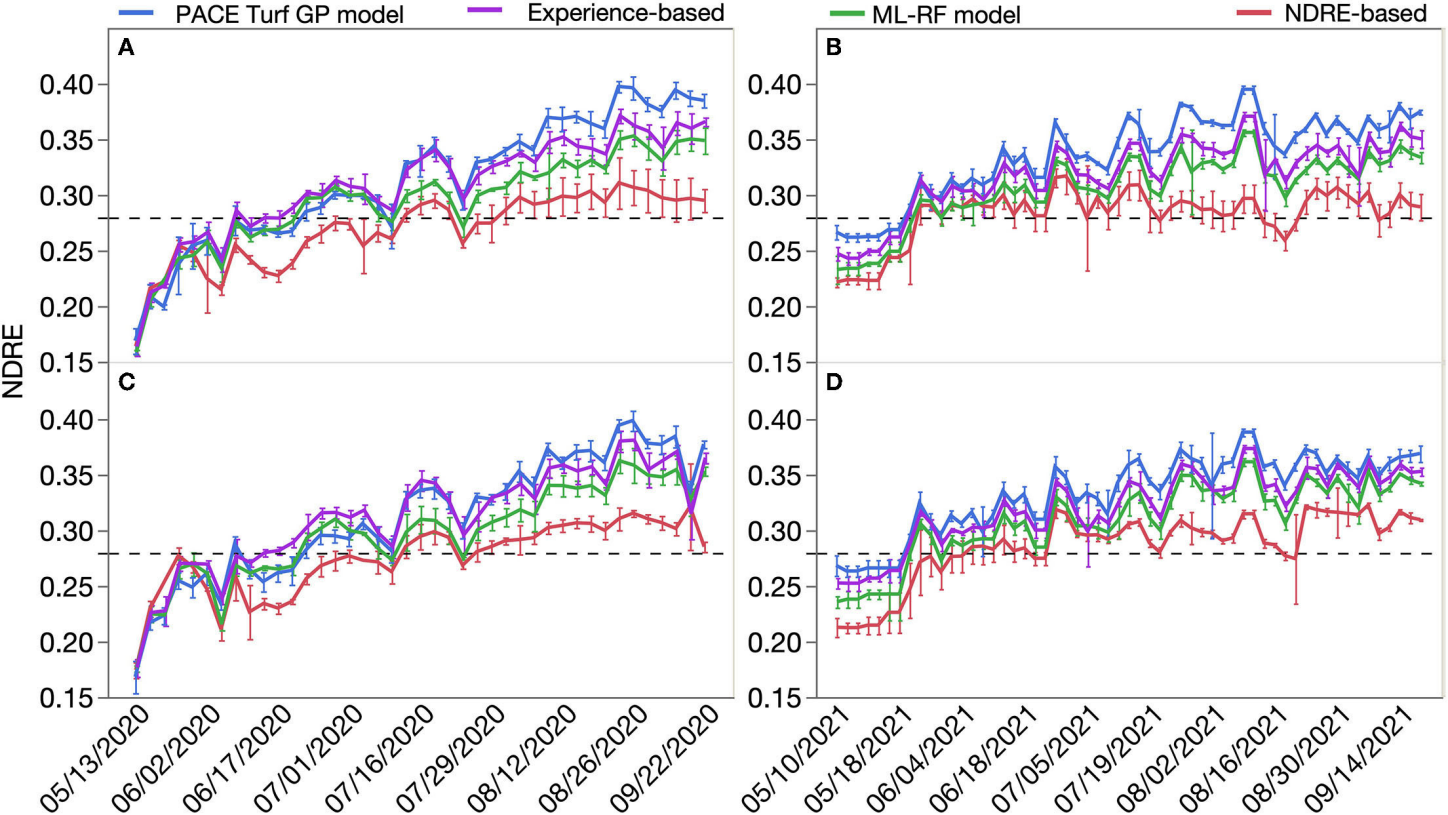
NDRE readings of creeping bentgrass response to four nitrogen (N) application strategies: the PACE Turf growth potential (GP) model, traditional N fertilization plan (experience-based), machine learning-random forest (ML-RF) model, and vegetative index (NDRE)-based strategy). **(A)** NDRE readings on root zone A in 2020. **(B)** NDRE readings on root zone A in 2021. **(C)** NDRE readings on root zone B in 2020. **(D)** NDRE readings on root zone B in 2021. The dashed line is the NDRE decision threshold of 0.28. Error bars indicate standard deviation.

The 2-year creeping bentgrass growth response to the four N application strategies and corresponding N fertilizer inputs is presented in Figure 2. Generally, there was greater clipping production when the turfgrass received greater N fertilization rates. In this study, the PACE Turf GP model resulted in the greatest amount of N fertilizer applied, followed by the experience-based method. The ML-RF model recommended the third most N fertilizer inputs, and creeping bentgrass clipping production from that method was also lower than the previous two N fertilization strategies. The NDRE-based strategy resulted in the least N fertilizer use and lowest clipping production on both root zones in both years. The creeping bentgrass growth response to four N application plans on both root zones had a similar trend, as N is one of the major contributions to turfgrass growth; however, there was a different growth pattern in each year, likely as a result of the different weather and soil conditions (i.e., temperature, moisture) in each year. Interestingly, creeping bentgrass clipping yield responses to the four N application strategies were similar during the first 5 weeks of the experiment even though different N fertilization rates were applied during this time. Turfgrass growth showed a delayed response to N fertilization. Creeping bentgrass NDRE readings under the four N fertilization treatments are presented in Figure 3. Similar to the growth response, plots that received N fertilizer recommendation following the PACE Turf GP model had the highest NDRE readings, followed by the experience-based method, ML-RF model, and the NDRE-based N fertilization strategy. The trends in NDRE readings on both greens and both years were similar, which implied that N fertilizer inputs were the primary driver of differences in NDRE readings. At the end of each season, PACE Turf GP model method had ~20% greater NDRE compared to the NDRE-based method, which represented the highest and lowest N fertilization amounts. The difference in the season-end NDRE readings between the PACE Turf GP model and ML-RF model was ~10%, and the difference between the PACE Turf GP model and the traditional N fertilization strategy was ~5%. Additionally, although there was no overall significant difference in creeping bentgrass clipping yield among the four N treatments in the first 5 weeks of the field experiment, there were significant differences in NDRE readings which demonstrated that N fertilization had an immediate impact on creeping bentgrass canopy characteristics. The creeping bentgrass readings were above the reference reading (0.28) among the four N treatments more times in 2021 than in 2020.

The 2-year mean creeping bentgrass clipping yield, NDRE, and turf quality response to four N treatments, as well the 2-year cumulative N fertilizer usage, clipping yield, and NUE, are presented in Table 2. There was no significant difference in clipping yield, NDRE, and turfgrass quality between the two root zones within the same N treatment. Turfgrass that received N treatments followed by the PACE Turf GP model produced significantly higher clipping yield, NDRE readings, and turf visual quality. The experience-based model produced the second most clipping yield, NDRE, and turf quality, followed by the ML-RF model and the NDRE-based N fertilization plan.

**Table 2:** The 2-year mean creeping bentgrass clipping yield, NDRE, and turfgrass quality responses to four nitrogen (N) application strategies on two research putting greens.

**Root zone ID**	**N app. strategies**	**Clipping** **(g m^−2^ d^−1^)**	**NDRE**	**Turf quality^†^**	**Sum of N fertilizer^¤^ (kg ha^−1^ 2yrs^−1^)**	**Sum of clipping** **(g m^−2^ 2yrs^−1^)**	**NUE^μ^ (%)**
A	PACE Turf GP^Θ^	1.63 a^‡^	0.328 a	7.6 a	281	297.5 a	33.1 d
	Experience^β^	1.38 b	0.315 b	7.4 ab	190	251.6 b	39.3 bc
	ML-RF approach^α^	1.20 c	0.302 c	7.2 b	136	216.7 c	44.6 b
	NDRE-based^Γ^	1.02 d	0.277 d	6.1 c	80	184.2 d	60.0 a
B	PACE Turf GP	1.62 a	0.326 a	7.5 a	281	288.9 a	32.5 d
	Experience	1.32 b	0.318 b	7.4 ab	190	240.1 b	37.8 cd
	ML-RF approach	1.17 c	0.306 c	7.2 b	142	213.9 c	43.2 bc
	NDRE-based	0.96 d	0.282 d	6.2 c	70	174.9 d	65.4 a

Θ *PACE Turf growth potential model-guided N application strategy.*

β *Traditional N application plan.*

α *Machine learning (random forest) growth model-guided N application strategy.*

Γ *Turfgrass vegetative index (NDRE)-based N application strategy.*

‡ *Within each column, means sharing the letter are not statistically different according to Fisher's protected LSD test (α = 0.05).*

† *Turf quality is evaluated on a scale from 1 to 9 where 1 represents completely dead turf, 6 represents the minimally acceptable quality, and 9 represents ideal turfgrass quality.*

¤ *Overall, N fertilizer use on the research plots in this study in 2020 and 2021.*

μ *NUE, nitrogen use efficiency, calculated by (N uptake by plant-N uptake by plant from non-fertilizer control plot)/2-year N fertilizer applied*.

The 2-year cumulative clipping yield from the non-fertilized control treatments on root zones A and B was 64.9 and 60.4 g m^−2^ 2 yrs^−1^, respectively. NUEs were highest (near 70%) on both root zones receiving N fertilization according to the NDRE-based method. NUEs were around 45% on the root zones following the ML-RF N fertilization method and were about 40% following the experience-based method. NUEs were lowest when using PACE Turf GP model method (34%). The PACE Turf GP model and experience-based methods do not customize N fertilization recommendations on different root zones, whereas the ML-RF model and the NDRE-based method are able to account for root zone properties. The experience-based method resulted in 32% less N fertilizer than the GP method, and the ML-RF model applied 52 and 49% less N fertilizer on root zones A and B, respectively, than the GP method. The NDRE-based method resulted in 72 and 75% less N fertilizer on root zones A and B, respectively, than the GP method.

**Figure 4:**
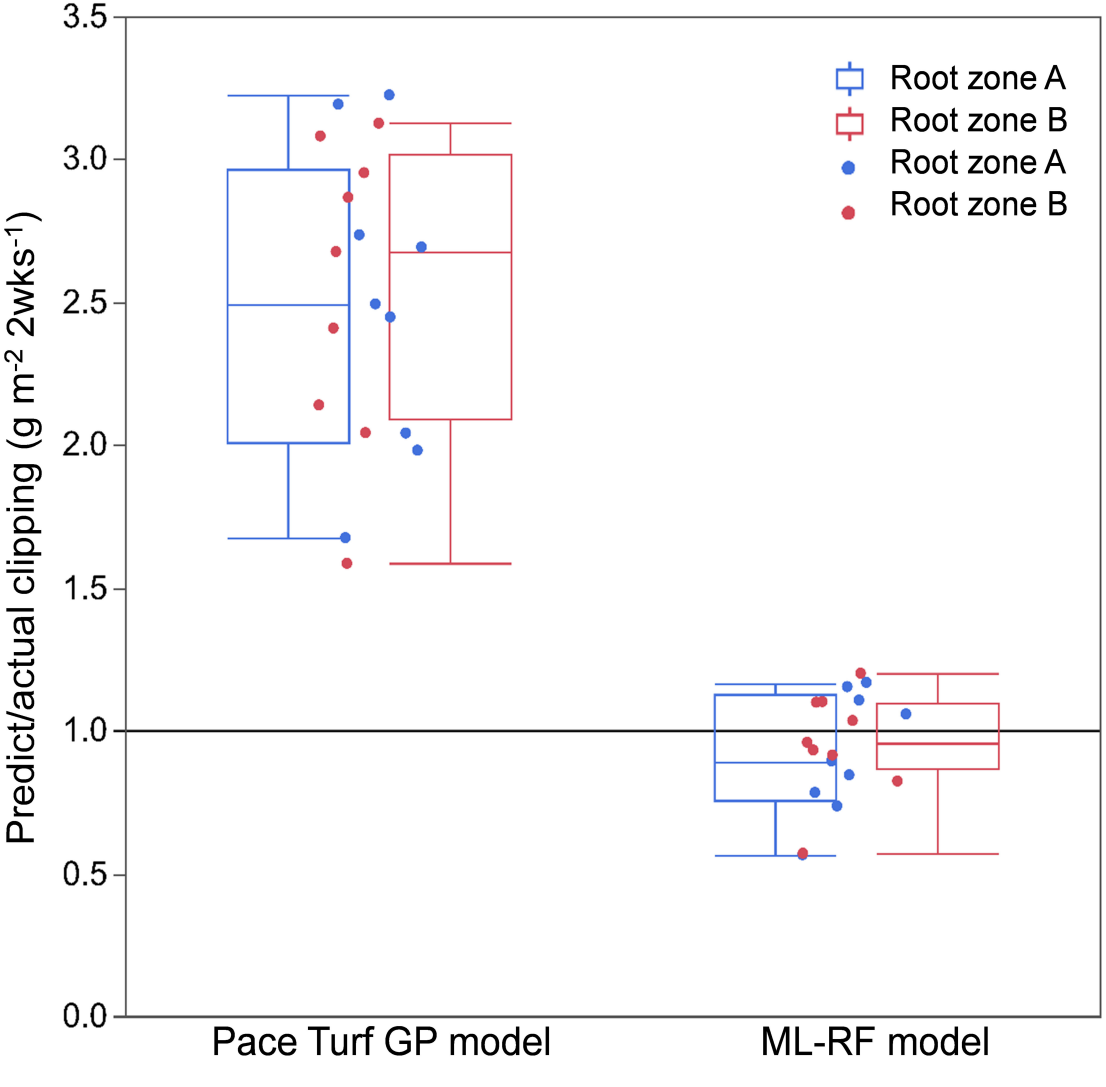
Creeping bentgrass growth prediction accuracy of PACE Turf growth potential (GP) model and machine learning-random forest (ML-RF) model. Blue boxes and dots represent data collected on root zone A and red boxes and dots represent data collected on root zone B. The boxplots were set at maximum, 75th (the upper quartile), median, and 25th (the lower quartile), and minimum.

The PACE Turf GP model and ML-RF model were the two approaches to making N application decisions based on turfgrass growth rate. Figure 4 presents the prediction accuracy of 2-week cumulative clipping yield using the two models. The ratio of predicted and observed clipping yield was near 1 with the ML-RF model, and therefore, it accurately predicted creeping bentgrass clipping yield on both research greens. The ratio of predicted to actual clipping yield with the PACE Turf GP method was much larger than ML-RF model ratio (2.5 and 2.7 for research root zones A and B, respectively). The range of the ratio when using the PACE Turf GP model spanned 1.7 to 3.2 for root zone A and 1.6 to 3.1 for root zone B. The range of the ratio when using ML-RF model spanned 0.6 to 1.2 for both root zones, and if the first clipping collection event of each year was excluded, the range of the ratio improved to 0.8 to 1.2.

**Figure 5:**
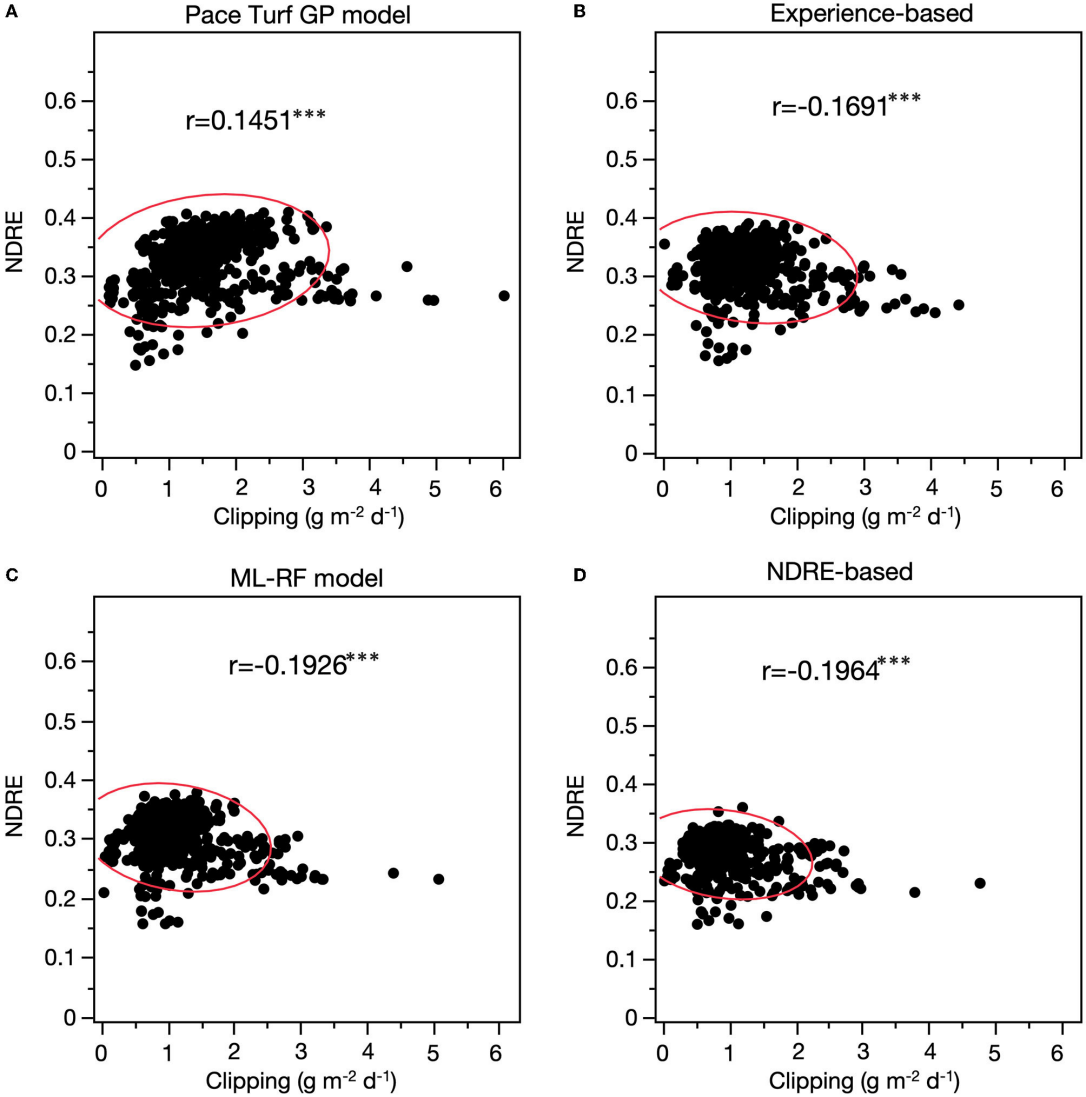
Pearson correlation between vegetative index (NDRE) and corresponding creeping bentgrass clipping yield, where the turfgrass was fertilized using different strategies **(A)**. PACE Turf GP model: using the PACE Turf growth potential model; **(B)** experience-based: traditional N fertilization plan that is based on turfgrass quality and manager's experience; **(C)** ML-RF model: machine learning-random forest growth prediction model; **(D)** NDRE-based: vegetative index (NDRE)-guided N fertilization. The red lines illustrate the 95% prediction.

Among four treatments, there were significant but weak correlations between daily clipping yield and NDRE across 2 years (Figure 5), where the correlation coefficient ranged from −0.20 to 0.15. Interestingly, the PACE Turf model had a weak positive correlation between daily clipping yield and NDRE, whereas the three other treatments all appeared to have negative correlation between daily clipping yield and NDRE.

## Discussion

Averaged over two seasons, we recovered 34–71% of applied N in clippings among the four N application strategies. According to Miltner et al. (1996), about 35% of applied N fertilizer was recovered in Kentucky bluegrass (*Poa pratensis*) clippings, and the majority of the rest of the applied N was immobilized in thatch and soil. We did not attempt to quantify the fate of the remaining fertilizer N in this study, and the long and short-term fate of the un-recovered N requires further investigation, but lower N recovery does not necessarily imply N leaching losses, and greater recovery of applied N is generally desirable assuming performance goals are being met.

The ML-RF model more accurately predicted creeping bentgrass clipping yield than the GP method and therefore more accurately estimated N removal from mowing. During the 2-year field experiment in Verona WI, USA, the average air temperature during the study period was 18.7°C in 2020 and 20.1°C in 2021 which both were near the optimum temperature for cool-season grass. Therefore, the N fertilization rates applied based on the GP model maintained the turfgrass growth at or close to the maximum growth rate that we selected based on the 2019 growing season (not necessarily the genetic maximum growth potential of the grass). A lower fertilization rate would have resulted with this method if we had selected a lower “maximum” growth rate. Therefore, calibration of the PACE Turf GP model may improve its utility as a decision support tool for nutrient applications. The GP model is simpler to use than the ML-RF model and therefore may have a wider reach.

Vegetation indices such as NDRE and NDVI have been evaluated as methods to guide N fertilization or quantify turfgrass response to fertilization (Kruse et al., 2006; Bremer et al., 2011a; Lee et al., 2011; López-Bellido et al., 2012; Inguagiato and Guillard, 2016; Guillard et al., 2021). Our study demonstrated that there was a weak correlation between creeping bentgrass clipping yield and NDRE. This implied that NDRE would also have a weak correlation with turfgrass growth and N uptake. Interestingly, we observed a weak positive correlation between daily clipping yield and NDRE when the N rate was based on the PACE Turf GP model (relatively higher N), whereas the correlation became negative once the N application rates were lower (experience-based, ML-RF model, and NDRE-based methods). Studies have concluded that NDRE has much stronger correlations with some turfgrass growth characteristics such as turfgrass biomass (Marín et al., 2020) and turfgrass N status (Guillard et al., 2021). These studies applied a wider range of N rates which resulted in large variation in turfgrass growth. In this study, N fertilizer was applied at a relatively smaller range, and this could be the reason for the weak correlation between NDRE and turfgrass clipping yield.

Additionally, NDRE and other vegetative indices can be affected by many variables in the field, such as canopy density (Bremer et al., 2011b), turfgrass water status (Caturegli et al., 2020), plant colorants (Obear et al., 2017), and other stresses (Fenstermaker-Shaulis et al., 1997; Badzmierowski et al., 2019). Because these stresses can be independent of plant N status, using NDRE to make N fertilization decisions could be oversimplistic. In contrast, the machine learning growth prediction model was designed for sand-based greens and uses a simplified N cycle to make fertilizer recommendations. This method requires collecting and tracking clipping yield for calibration purposes and is only practical for golf course putting greens. For other parts of a golf course, such as fairways where it may not be practical to measure clipping yield and are under less stress than putting greens, using NDRE or other vegetative indices to guide N application decision could be more appropriate.

The ML-RF model in this study relied on the previous years' clipping yield as well as the current year's weather data, management practices, and vegetative indices. It was able to provide site-specific N recommendations for turfgrass planted in different soil conditions, different micro-climates, and under different management practices. The model also resulted in reduced N input compared to the traditional experience-based method and resulted in acceptable turfgrass quality. Similar studies (Engel et al., 1999; Long et al., 2000) also employed precision N management by monitoring and estimating N and as a result increased crop quality, yield, and economic profit. The ML-RF N application strategy proposed in this study is anchored in the N cycle and allows turfgrass managers to adjust the target clipping yield or clipping volume to meet different performance goals.

Ericsson et al. (2012, 2013) concluded that 3.1 to 3.5% leaf N concentration was sufficient to achieve a good turfgrass color and quality in most turfgrasses, including bentgrass and fescues (*Festuca spp*.). They also concluded that turfgrass with 60% of maximum growth would be sufficient to produce good turfgrass playing quality. The drawbacks to such a generalized approach include the need to frequently send leaf tissue to the laboratory for leaf N analysis, knowledge of the maximum growth rate, and measuring turfgrass yield to compare against that maximum growth rate. In contrast, the ML-RF model could help turfgrass managers to make clipping yield predictions based on weather data and management practice input without spending time on clipping collection aside from the calibration period. However, the ML-RF model does not yet exist in a user-friendly graphical interface, so one would need to be created for it to become widely used. A user-friendly decision support tool would be able to automatically process the data input without the need for knowledge of coding from end-users, additionally, it would predict turfgrass clipping yield of each golf course greens for the next weeks depending on the management practices, environment, and weather data input. The decision support tool would provide N fertilizer recommendations for the next N application event based on predicted clipping yield.

Many turfgrass managers of golf courses are beginning to recognize the benefits of regularly measuring grass growth by tracking clipping volume of golf course putting greens, and adjusting N fertilization based on the collected clipping. Precision N fertilization application has the potential to provide economic (i.e., reduced N fertilization input and other resource inputs, such as labor and energy) and environmental benefits (i.e., reduced N leaching and gaseous losses). However, there is not enough research, including economic research and environmental assessment, to evaluate precision N management and compare with the experience-based method which has been widely used. A better understanding of the economic and environmental outcomes from precision N management could help turfgrass managers choose optimized N fertilization methods.

## Conclusion

Sustainable turfgrass systems integrate the goals of environmentally friendly and economic profitability. Among other resource inputs on turfgrass systems, the efficient and effective use of N fertilizer is one of the main drivers for improving sustainability, and proper N fertilization is in the economic interest of those in the golf and turfgrass industry. Our proposed precision N management attempts to help golf courses optimize N fertilizer use while maintaining quality standards on putting greens. Several N fertilizer management strategies were evaluated on golf course creeping bentgrass putting greens. The results demonstrated that a ML-RF method was able to significantly reduce N fertilizer usage and increase N use efficiency while maintaining high-quality turfgrass relative to the traditional method for fertilization. Whereas, the ML-RF and PACE Turf GP methods were both based on turfgrass growth predictions, the ML-RF method was able to more accurately estimate clipping removal and therefore may be useful for helping turfgrass managers to tie N fertilization decisions to the N cycle, rather than simply basing decisions on experience and visual observations.

## Data Availability Statement

The original contributions presented in the study are included in the article/supplementary material, further inquiries can be directed to the corresponding authors.

## Author Contributions

QZ and DS designed the study and conducted field experiments. QZ performed data analysis, built the prediction model, and wrote the manuscript. DS provided critical insights, edited, and revised the manuscript. Both authors reviewed the manuscript and agreed with the submission.

## Funding

This work was partially funded by the United States Golf Association (Grant #: 2019-10-680), the Wisconsin Golf Course Superintendents Association, and the Wisconsin Turfgrass Association.

## Conflict of Interest

The authors declare that the research was conducted in the absence of any commercial or financial relationships that could be construed as a potential conflict of interest.

## Publisher's Note

All claims expressed in this article are solely those of the authors and do not necessarily represent those of their affiliated organizations, or those of the publisher, the editors and the reviewers. Any product that may be evaluated in this article, or claim that may be made by its manufacturer, is not guaranteed or endorsed by the publisher.
